# Iron oxide nanocatalyst with titanium and silver nanoparticles: Synthesis, characterization and photocatalytic activity on the degradation of Rhodamine B dye

**DOI:** 10.1038/s41598-020-59987-0

**Published:** 2020-02-20

**Authors:** Pâmela Cristine Ladwig Muraro, Sérgio Roberto Mortari, Bruno Stefanello Vizzotto, Gabriela Chuy, Cristiane dos Santos, Luís Fernando Wentz Brum, William Leonardo da Silva

**Affiliations:** 1 Universidade Franciscana - Programa de Pós-Graduação em Nanociências Rua Silva Jardim no 1323, Santa Maria, RS 97010-491 Brasil; 2 Instituto de Química – Universidade Federal do Rio Grande do Sul Av. Bento Gonçalves no 9500, Porto Alegre, RS 91501-970 Brasil

**Keywords:** Pollution remediation, Environmental, health and safety issues

## Abstract

Nowadays, there is a growing concern about the environmental impacts of colored wastewater. Thus, the present work aims the synthesis, characterization and determination of photocatalytic activity of iron oxide (Fe_2_O_3_) nanocatalyst, evaluating the effect of hybridization with titanium (TiNPs-Fe_2_O_3_) and silver (AgNPs-Fe_2_O_3_) nanoparticles, on the degradation of Rhodamine B dye (RhB). Nanocatalysts were characterized by XRD, SEM, TEM, FTIR, N_2_ porosimetry (BET/BJH method), zeta potential and DRS. Photocatalytic tests were performed in a slurry reactor, with the nanocatalyst in suspension, using RhB as a target molecule, under ultraviolet (UV) and visible radiation. Therefore, the photocatalytic activity of the nanocatalysts (non-doped and hybridized) was evaluated in these ideal conditions, where the AgNPs-Fe_2_O_3_ sample showed the best photocatalytic activity with a degradation of 94.1% (k = 0.0222  min^−1^, under UV) and 58.36% (k = 0.007  min^−1^, under visible), while under the same conditions, the TiO_2_-P25 commercial catalyst showed a degradation of 61.5% (k = 0.0078  min^−1^) and 44.5% (k = 0.0044  min^−1^), respectively. According with the ideal conditions determined, reusability of the AgNPs-Fe_2_O_3_ nanocatalyst was measured, showing a short reduction (about 8%) of its photocatalytic activity after 5 cycles. Thus, the Fe_2_O_3_ nanocatalyst can be considered a promising catalyst in the heterogeneous photocatalysis for application in the degradation of organic dyes in aqueous solution.

## Introduction

Dyes are substances with high application potential in the most diverse areas, mainly to color the final products of textile, precious stones, leather, paper, plastics and food. For example, it is estimated that there are more than 100,000 synthetic dyes, with an annual production of more than 700,000 tons worldwide, generating a significant amount of wastewater^1^. In addition, these colored waters are characterized by complex aromatic compounds, making their biodegradation difficult, becoming an environmental liability. Thus, advanced processes are needed to promote their correct treatment in order to meet environmental norms and legislation^2^.

In this context, the Advanced Oxidative Processes (AOPs), highlighting the heterogeneous photocatalysis, becomes an attractive alternative, since they are technologies with potential to oxidize a great variety of complex organic compounds^3^, using a highly oxidant and less selective species (the hydroxyl radical, ^•^OH), capable of mineralize many organic compounds^4^. Thus, the heterogeneous photocatalysis involves the photoactivation of a semiconductor (catalyst), under visible or ultraviolet radiation, with energy equal to or greater than band gap energy^5^, promoting oxy-reduction reactions on the catalytic surface and thus the degradation of organic pollutants.

Among the most used catalysts are titanium dioxide (TiO_2_), cadmium sulfide (CdS), zinc oxide (ZnO), zinc sulphide (ZnS), tungsten trioxide (WO_3_), tin dioxide (SnO_2_) and iron oxide III (Fe_2_O_3_)^6^. However, these catalysts generally present a low specific surface area and porosity, limiting intraparticle diffusion of organic pollutants, compromising its photocatalytic activity^7–9^.

Nanocatalyst has emerged as an alternative to increase catalytic efficiency, since it has advantages over commercial catalysts, such as higher specific surface area and porosity, making them with great potential application in heterogeneous photocatalysis^10^. One of the strategies used to increase the photocatalytic activity of nanocatalysts is the usage of hybridization with noble metal and metals, in order to reduce the recombination between photoelectrons/holes pairs and reducing the energy required to its photoactivation, allowing its application to visible radiation^11–16^. In addition, Rhodamine B dye (RhB) (C_28_H_31_N_2_O_3_Cl), a highly water soluble organic cation dye, belongings to the class of xanthenes, whose contact with humans can cause irritation to the skin, airways and eyes^17^. Moreover, it presents the chromophoric groups (−C=C −/− C=N−), as well as a characteristic carcinogenicity and neurotoxicity activity.

In this context, the present work aims the synthesis, characterization and determination of photocatalytic activity of Fe_2_O_3_ nanocatalyst, hybridized with titanium nanoparticles (TiNPs) and silver nanoparticles (AgNPs) on the degradation of Rhodamine B (RhB) dye, under UV and visible radiation.

## Materials and Methods

### Synthesis of the Fe_2_O_3_ nanocatalyst

The synthesis of iron oxide nanocatalyst followed the chemical precipitation by sodium borohydride method, according to the literature^18^. Sodium borohydride (NaBH_4_, 0.2  mol  L^−1^, Neon, PA) and ferric chloride hexahydrate (FeCl_3_∙H_2_O, 0.05  mol  L^−1^, Synth, PA) were mixed for 30  minutes, under magnetic stirring (250  rpm). After, the synthesized nanoparticles were vacuum filtered and washed with deionized water and diluted ethanol (~5%). Parameters such as pH (≈ 7), reagents concentrations, stirring speed, reaction time and temperature (23 ± 0.5 °C) were kept constant in order to avoid influence on the composition and properties of nanocatalyst.

### Synthesis of nanoparticles (NPs)

For silver nanoparticles (AgNPs), 75  mL of sodium borohydride solution (0.002  mol  L^−1^, Neon, PA) was added, in ice bath, under for magnetic stirring for 10–15  min. with 25  mL of a silver nitrate solution (0.001  mol  L^−1^, Synth, PA) (rate of 1 drop s^−1^), forming silver nanoparticles. It is noteworthy that titanium nanoparticles were commercially purchased (TiO_2_, Evonik Aeroxide P25).

### Synthesis of hybridized nanocatalyst

For hybridization of Fe_2_O_3_ nanocatalyst, the impregnation methodology was used with TiNPs and AgNPs, according to the literature^19^. Samples were magnetic stirring at room temperature for 90  min, after calcined at 450 °C (heating rate 10 °C min^−1^) for 4  hours. Finally, the granulometry was standardized with milling and sieving (# 12). Hybridized nanocatalysts with NPs were labeled as TiNPs-Fe_2_O_3_ and AgNPs-Fe_2_O_3_, respectively.

### Characterization of nanocatalyst

X-ray diffraction (XRD) was used to determine the crystallinity of the samples in a Bruker D2 Advance diffractometer with a copper tube (Kα = 1.5418 Ǻ) in the range of 5° to 70°, with tension acceleration and applied current of 30  kV and 30  mA, respectively.

For zeta potential (PZ), Malvern-Zetasizer® model nanoZS (ZEN3600, UK) with closed capillary cells (DTS 1060) (Malvern, UK) was used to measure the zeta potential values of the samples.

N_2_ porosimetry was used to evaluate the textural properties of specific surface area (S_BET_) and porosity (pore diameter – Dp and pore volume – Vp) using in an equipment Gemini VII 2375 Surface Area Analyzer Micromeritics® and BET/BJH Methods (P Po^−1^ = 0.05–0.35).

Band gap energy was determined by diffuse reflectance spectroscopy at UV radiation (UV DRS) using a Varian Cary Scan Spectrophotometer with DRA-CA-301 accessory (Labsphere) coupled in the diffuse reflectance mode to determine the energy band gap by means of the Kubelka–Munk function with scans ranged from 200 to 600  nm.

Scanning electron microscopy (SEM) and Transmission electronic microscopy (TEM) were used to morphologically characterize the nanocatalysts (Fe_2_O_3_, TiNPs-Fe_2_O_3_ and AgNPs-Fe_2_O_3_) using a JSM5800 (JEOL) and JEM 1200 Exll (JEOL) microscope, respectively. Moreover, the samples were coated with a thin layer of conductive gold by a sputtering technique.

### Effect of the photolysis and adsorption on the RhB degradation

To evaluate the influence of adsorption on degradation of RhB dye, were carried out preliminary tests (time = 90  min, pH ≈ 4.03, [Fe_2_O_3_] = 0.7  g  L^−1^ and [RhB] = 20  mg  L^−1^) in dark (without irradiation) conditions to establish the time required for the equilibrium of RhB molecule on nanocatalyst surface. Moreover, the effect of photolysis on RhB degradation was carried out by tests without catalyst, only under radiation with RhB dye solution (20  mg  L^−1^, 61.8  W  m^−2^ for UV irradiation and 202  W  m^−2^ for visible irradiation and time = 60  min).

### Photocatalytic activity

Photodegradation tests were carried out using a solution of RhB, as the target molecule, and nanocatalysts in suspension (slurry). Tests were performed in two stages: (a) dark stage (absence of radiation), where adsorption/desorption equilibrium of the target molecule occurred on the surface of the nanocatalyst, with a duration of 60  minutes, and (b) a photocatalytic reaction step (with visible or UV radiation) with duration of 120  minutes (collections at predetermined times of (0, 5, 15, 30, 45, 60, 75, 90, and 120  minutes). Then the samples were centrifuged (Cientec CT-5000R refrigerated centrifuge) for 20  minutes with a rotation of 5,000  rpm and finally diluted (1:10  v/v).

Moreover, the absorbance measurements of solutions collected during reactions were carried out in a double-beam spectrophotometer (Varian, Cary 100) with a halogen lamp at the wavelength characteristic of RhB (λ = 553  nm)

### Kinetic study of RhB degradation

In order to determine the specific reaction rate (k), a kinetic study of RhB dye degradation, under UV and visible radiation over time, was carried out according to the classic heterogeneous kinetic model (pseudo first-order model) (Eqs. 1 and 2)^20,21^:

1 $$-{r}_{i}=-\,\frac{d{C}_{i}}{dt}=\frac{{k}_{S}\cdot K\cdot {C}_{i}}{1+K\cdot {C}_{i}}$$

Or:

2 $$\mathrm{ln}(\frac{{C}_{io}}{{C}_{i}})={k}_{S}\cdot K.t=k.t\,{\rm{or}}\,{C}_{i}={C}_{io}\cdot {e}^{-k\cdot t}$$

### Experimental designs

Central Rotatable Composite Design (CRCD) (Statistic 8.0, StatSoft, Tulsa, OK, USA) symmetrical and of second order was used to determine the ideal reaction conditions (pH, [RhB] and [Fe_2_O_3_]) for the degradation of RhB dye, constituted of a factorial 2^3^, with 8 tests, 3 central points, and 6 axial points, totalizing 17 experiments, according to Table 1.

**Table 1:** Factorial planning matrix 2^3^ for Fe_2_O_3_ nanocatalyst.

Order	[RhB] (mg L^−1^)	[Fe_2_O_3_] (g L^−1^)	pH
(−1.68)	5	0.5	2
(−1)	24.25	1.41	4.03
0	52.5	2.75	7
(+1)	80.74	4.08	9.97
(+1.68)	100	5	12

## Results and Discussion

### Characterization of nanocatalysts

Table 2 shows the results of N_2_ porosimetry, DRS and zeta potential characterization.

**Table 2:** Surface area (S_BET_), pore volume (Vp), pore diameter (Dp), band gap energy (Eg) and zeta potential (PZ) of the synthesized nanocatalysts.

Samples	S_BET_ (m² g^−1^)	Vp (cm³ g^−1^)	Dp (nm)	Eg (eV)	ZP (mV)
TiO_2_ (P25)	56	0.07	4.8	3.2	−24.0
Fe_2_O_3_	158	0.93	22.6	2.2	−13.3
TiNPs- Fe_2_O_3_	304	0.02	2.9	2.0	−11.5
AgNPs- Fe_2_O_3_	505	1.60	3.5	1.8	−10.2

According to Table 2, AgNPs- Fe_2_O_3_ showed the highest S_BET_ (505  m² g^−1^), while TiNPs- Fe_2_O_3,_ (304  m² g^−1^), while Fe_2_O_3_ (158  m^2^  g^−1^) and TiO_2_ (P25) (56  m^2^  g^−1^) nanocatalysts. In relation to pore volume (Vp), AgNPs-Fe_2_O_3_ showed the highest volume (1.6  cm³ g^−1^). In addition, according to pore diameter (Dp), all nanocatalysts (non-doped and doped) showed mesoporous characteristics^22^. For heterogeneous photocatalysis, surface area and porosity (pore volume and diameter) are factors that directly affect photocatalytic performance, since they affect the adsorption of the target molecule and oxy-reduction reactions for hydroxyl radical formation^23,24^.

The band gap of Fe_2_O_3_, TiNPs-Fe_2_O_3_ and AgNPs-Fe_2_O_3_ were found between 2.2; 2.0 and 1.8  eV, respectively. Thus, nanoparticles (NPs) promoted a reduction in conduction and valence bands, compared to Fe_2_O_3_ nanocatalyst, generating a decrease in the energy required for photoactivation of the hybridized nanocatalysts and shifting the application of nanocatalysts to the visible region of radiation^25^.

Moreover, all samples showed a negative charge surface potential (−10.25 to −13.30  mV), according to zeta potential, indicating a charge compatibility, since RhB dye is characterized by its cationic nature^26,27^, increasing the RhB adsorption capacity on the catalytic surface and thus a possible better photocatalytic activity.

**Figure 1:**
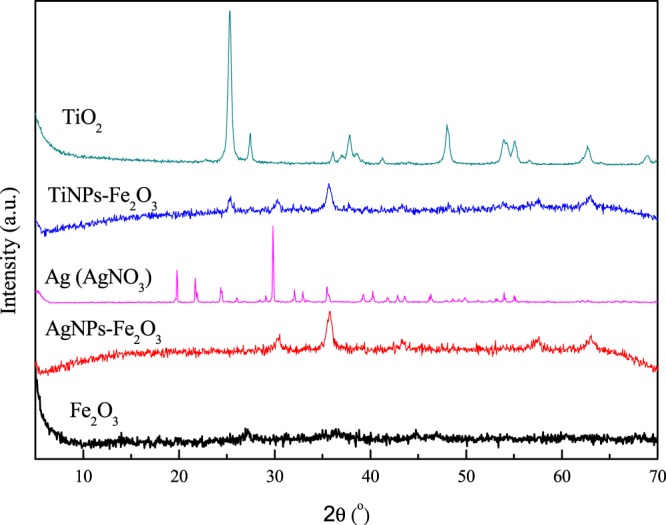
X-ray diffraction of nanocatalysts, (Fe_2_O_3_, TiNPs-Fe_2_O_3_ and AgNPS-Fe_2_O_3_) and standard XRD (TiO_2_ and AgNO_3_).

Figure 1 shows the X-ray diffractograms of the synthesized samples (without and with NPs hybridization).

According to Fig. 1, it is possible to identify the characteristic peaks of the photo phase of TiO_2_ (anatase) at 25.2°; 37.8°; 48.2°; 53.8° e 55.0° corresponding respectively to the plans (101), (004), (200), (105), e (211); some rutile diffraction peaks are still observed, the main one being 2θ = 27.4° which corresponds to the plane (110), according to JCPDS (Code 21–1272)^28^. Moreover, the silver nitrate X-ray diffraction pattern (AgNO_3_) showed the characteristic peaks, with their respective crystalline planes, at 30.2° (001), 35.6° (111), 43.6° (200) and 62.3° (220)^29^. In addition, it was possible to identify peaks of Fe_2_O_3_ oxide (33.2°, 35.6°, 40.9°, 54.1°, 62.5° and 64.1°) (JCPDS - Code 01–1053)^30,31^. Thus, the hybridization process with the NPs did not promote the formation of new peaks, as well as titanium and silver characteristic peaks were identified on Fe_2_O_3_ sample, indicating a successful hybridization.

**Figure 2:**
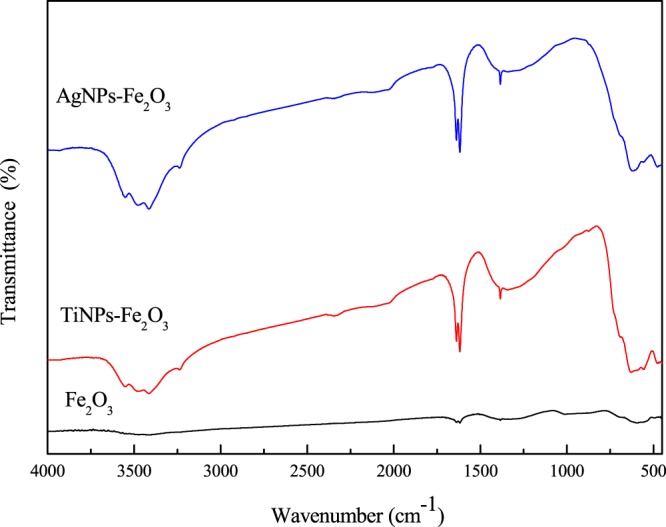
FTIR spectrum of nanocatalysts.

FTIR analysis was used as a qualitative analysis technique to determine the functional groups present in the synthesized materials, according to Fig. 2.

According to Fig. 2, iron oxide showed at 3394  cm^−1^ strip, is attributed to stretch vibrations (ν), while the 1620 cm^−1^ strip is attributed to flexural vibrations (δ) due to water adsorbed on the surface of the iron oxide nanoparticles^32^. The band observed at 611  cm^−1^ corresponds to the stretching vibrations of M_Th_-O-M_Oh_, where M_Th_ and M_Oh_ correspond to iron occupying tetrahedral and octahedral positions, respectively. TiNPs showed at 590  cm^−1^ due to the vibration of the TiO-O bond^33,34^.

**Figure 3:**
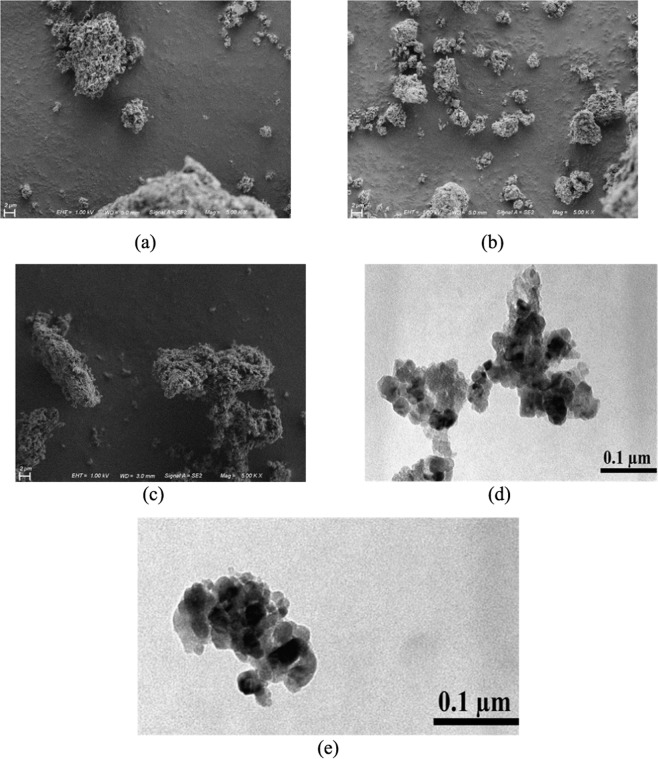
SEM micrographs for the samples: (**a**) Fe_2_O_3_, (**b**)TiNPs- Fe_2_O_3_ and (**c**) AgNPs- Fe_2_O_3_, and TEM micrographs for the (**d**) TiNPs-Fe_2_O_3_ and (**e**) AgNPs-Fe_2_O_3_.

Figure 3 shows SEM micrographs of the nanocatalysts (a) Fe_2_O_3_, (b) TiNPs-Fe_2_O_3_ and (c) AgNPs-Fe_2_O_3_, while Fig. 3(d,e) show the TEM micrographs of the TiNPSs-Fe_2_O_3_ and AgNPs-Fe_2_O_3_ samples, respectively. Then, it was possible to identify a heterogeneous surface with a random distribution of the TiNPs and AgNPs, with the formation of small clusters of NPs. Then, it can be explained through of the zeta potential (surface charge), since using NPs, the ZP showed a smaller between the nanoparticles, causing a lower dispersion, in relation to Fe_2_O_3_ nanocatalyst (with higher value of ZP)^33^. Thus, this greater dispersion of NPs tends to promote changes in the textural properties of nanocatalysts, such as an improve of the specific area and a greater number of active sites to conductive the RhB adsorption, directly affecting photocatalytic activity^34^. Therefore, TEM micrographs showed the presence of NPs (TiNPs and AgNPs) over Fe_2_O_3_ in spherical shape with a diameter around 3  nm.

### Adsorption and photolysis

Preliminary tests of the adsorption showed that the minimum time necessary for the adsorption equilibrium of RhB was 60  minutes, with an adsorption percentage of 4.71, 8.70 and 10.32% to Fe_2_O_3_, TiNPs-Fe_2_O_3_ and AgNPs-Fe_2_O_3_, respectively. The preliminary tests of photolysis indicated that only 14% (UV radiation) and 4% (visible radiation) of RhB were degraded after 60  minutes.

### Central rotatable composite design

**Figure 4:**
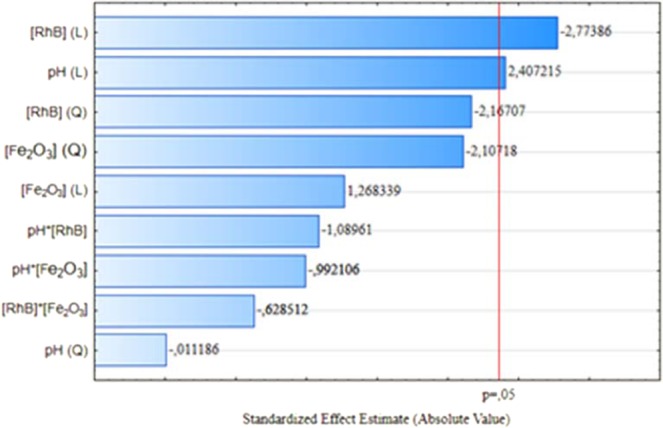
Pareto chart on the effect of the pH, [RhB] and [Fe_2_O_3_] on the degradation of RhB dye.

Figure 4 shows the Pareto chart used to evaluate the main effects of the central rotatable composite design (pH, [RhB] and [Fe_2_O_3_]). The reduced quadratic model (Eq. (3)) represents the Fe_2_O_3_ CRCD experiments, in which “y” represents the dependent variable (percentage of photocatalytic degradation after 120  minutes).

3 $$y=59.84+10.26.pH-11.82.[RhB]$$

According to Fig. 4, the RhB concentration showed a negative effect, thus with increasing RhB concentration occurs a reduction of the photocatalytic activity on RhB degradation occurs due to the reduction in the number of active sites available for adsorption on surface of Fe_2_O_3_ the nanocatalyst^35^. About the effect of pH, it has a positive effect on RhB degradation, since with the increase of the pH, the surface of Fe_2_O_3_ nanocatalyst is more deprotonated, increasing the compatibility of charges between RhB and Fe_2_O_3_ and thus increasing adsorption and photocatalytic activity^36^.

Therefore, after all the photocatalytic tests, the ideal conditions determined were 1.41  g  L^−1^ to Fe_2_O_3_ nanocatalyst, 24.25  mg  L^−1^ RhB concentration and pH = 9.97, which showed the greatest degradation of the 77.38%, after 120  minutes under UV radiation.

### Photocatalytic performance

The photocatalytic performance of nanocatalysts (Fe_2_O_3,_ TiNPs-Fe_2_O_3_ and AgNPs-Fe_2_O_3_) was evaluated by the photodegradation of RhB, under UV and visible radiation, using the ideal conditions.

**Figure 5:**
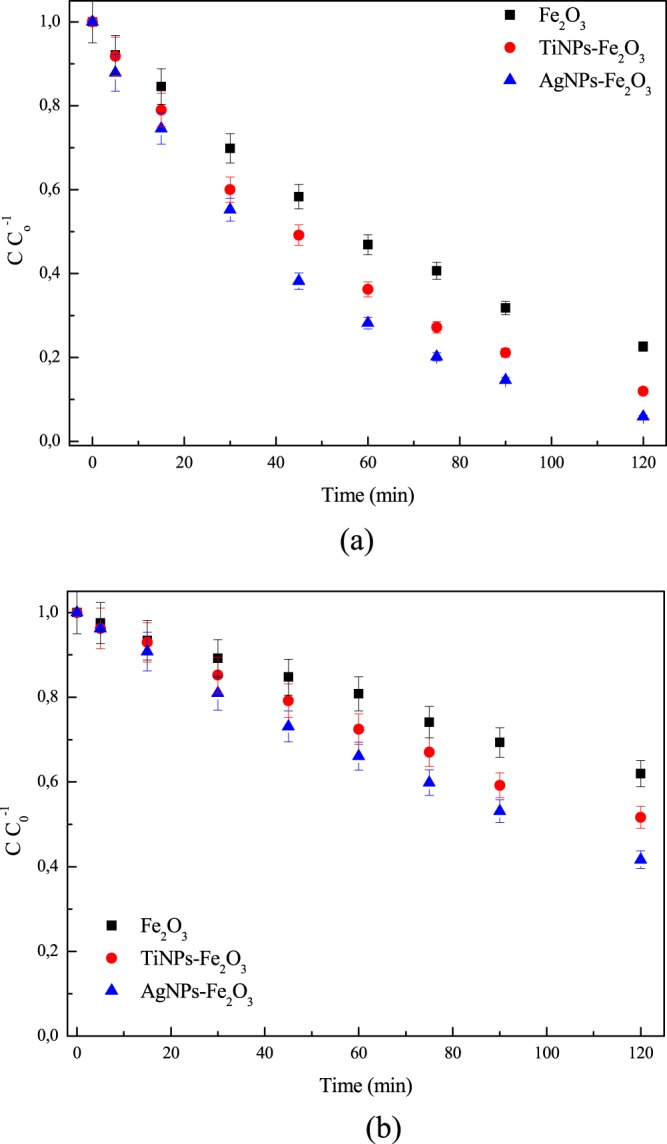
Photocatalytic activity of nanocatalysts (Fe_2_O_3_, TiNPs-Fe_2_O_3_ and AgNPs-Fe_2_O_3_) on RhB degradation under UV (**a**) and visible (**b**) radiation after 120  minutes ([catalyst] = 1.41  g  L^−1^, [RhB] = 24.25  mg  L^−1^, T = 30 °C, pH = 9.97, UV radiation of 61.8  W  m^−2^, visible radiation of 202  W  m^−2^ and error 5%).

After 120  min of UV radiation, 77.38% (k = 0.0124  min^−1^), 88.03% (k = 0.0173  min^−1^) and 94.10% (k = 0.022  min^−1^) of RhB were degraded using Fe_2_O_3_, TiNPs-Fe_2_O_3_ and AgNPs-Fe_2_O_3_, while 38.03% (k = 0.0039  min^−1^), 48.36% (k = 0.0055  min^−1^) and 58.36% (k = 0.0070  min^−1^) of RhB were degraded using Fe_2_O_3_, TiNPs-Fe_2_O_3_ and AgNPs-Fe_2_O_3_ under visible radiation, respectively, according to Fig. 5.

According to Fig. 5, the hybridization of Fe_2_O_3_ nanocatalyst with NPs (TiNPs and AgNPs) promoted an increase in the photocatalytic activity on the RhB degradation under UV radiation (13.8% using to TiNPs-Fe_2_O_3_ and 21.6% with AgNPs-Fe_2_O_3_) and visible radiation (27.2% - TiNPs-Fe_2_O_3_ and 55.4% - AgNPs-Fe_2_O_3_). This can be explained, due the fact that the nanoparticles (TiNPs and AgNPs) can act as electron traps facilitating the electron hole separation and subsequent transference of trapped electrons to the absorbed O_2_ acting as an electron acceptor on the surface of Fe_2_O_3_ nanocatalyst^35,36^. Thus, more molecules are adsorbed on the surface of hybridized nanocatalysts, enhancing the photo excited electron to the conduction band and simultaneously increasing the electron transfer to the adsorbed O_2_.

The power of the semiconductor material to act as a sensitizer and to enhance the photodegradation of the RhB is based on their electronic structure with filled valence bond and empty conduction bond^37^. The semiconductor photooxidation instigated the photocatalysis of RhB in solution, leaving the catalyst surface with a strong oxidative potential of an electron–hole pair (h^+^_VB_) (Eq. (4)), when photocatalyst was irradiated with higher energy than that of band gap energy (Eg), which allows the oxidation of the RhB molecule in a direct manner to the reactive intermediates (Eq. (5)).The hydroxyl radical (OH^•^), the exceptionally strong and a non-selective oxidant which is formed either by decomposition of water (Eq. (6)) or by reaction of hole along with hydroxyl ion (OH^−^) (Eq. (7)) is also responsible for degradation of phenol molecule. Leading to incomplete or complete mineralization of many organic molecules (Eq. (8))^38^.

4 $$NPs-F{e}_{2}{O}_{3}+h\nu \to NPs-F{e}_{2}{O}_{3}({e}_{CB}^{-}+{h}_{VB}^{+})$$

5 $$({h}_{VB}^{+})+RhB\to Rh{B}^{\bullet +}\to \,{\rm{oxidation}}\,{\rm{of}}\,{\rm{RhB}}\,{\rm{molecule}}$$

6 $$({h}_{VB}^{+})+{H}_{2}O\to {H}^{+}+H{O}^{\bullet }$$

7 $$({h}_{VB}^{+})+H{O}^{-}\to H{O}^{\bullet }$$

8 $$H{O}^{\bullet }+RhB\to {\rm{degradation}}\,{\rm{of}}\,{\rm{RhB}}\,{\rm{molecules}}$$

### Effect of AgNPs-Fe_2_O_3_ recycling

The effect of reuse of the AgNPs-Fe_2_O_3_ nanocatalyst under visible radiation (202  W  m^−^²) was evaluated in 5 times, according to Table 3.

**Table 3:** Effect of AgNPs-Fe_2_O_3_ nanocatalyst reuse on degradation of the RhB.

Cycle number	Degradation (%)
Cycle I (fresh nanocatalyst)	58.36
Cycle II	57.40
Cycle III	56.68
Cycle IV	55.73
Cycle V	54.39
Cycle VI	53.69

According to Table 3, the AgNPS-Fe_2_O_3_ nanocatalyst showed a photostability after five recycling processes, with a small decrease (about 8%) in the photocatalytic activity (58.36% to 53.69%), under visible radiation.

## Conclusion

According to the characterization and photocatalytic activity results, the hybridization process with NPs (TiNPs and AgNPs) caused positive changes in the properties of Fe_2_O_3_ nanocatalyst for heterogeneous photocatalysis, such as: reduction of band gap energy (2.2  eV to 2.0  eV - TiNPs-Fe_2_O_3_ and 1.8  eV - AgNPs-Fe_2_O_3_); increase of surface area (158  m² g^−1^ to 304  m² g^−1^ - TiNPs-Fe_2_O_3_ and 505  m² g^−1^ - AgNPs-Fe_2_O_3_) and increase in photocatalytic activity under UV and visible radiation. Therefore, nanoparticle hybridization on nanocatalysts is a great option to improve the photocatalytic performance for degradation of organic pollutants (such as dyes) by heterogeneous photocatalysis.
